# Environmental Regulation and Exports: Evidence from the Comprehensive Air Pollution Policy in China

**DOI:** 10.3390/ijerph18031316

**Published:** 2021-02-01

**Authors:** Hong Chen, Yang Xu

**Affiliations:** Economics and Management School, Wuhan University, Wuhan 430072, China; 00008567@whu.edu.cn

**Keywords:** environmental regulation, air pollution policy, export, pollution haven effect, China

## Abstract

The impact of environmental regulation has been an important topic. Based on the Chinese Custom Database and China City Statistical Yearbook, this paper investigates the effect of environmental regulation on export values and explores potential mechanisms and heterogeneous effects. Taking advantage of China’s first comprehensive air pollution prevention and control plan, the Air Pollution Control in Key Zones policy, as a quasi-natural experiment, we employ the difference-in-differences method to examine the causal relationship between environmental regulation and exports. We find the statistically significant and negative effect of environmental regulation on exports at the city level. Moreover, we find that the potential mechanism is the change in export values caused by firm entry and exit, especially by exiters, rather than the change in the number of exporting firms in the city caused by firm entry and exit. In addition, we find the heterogeneous effects of environmental regulation based on the differences of environmental policy across cities and the Broad Economic Categories classification.

## 1. Introduction

With the deterioration of the environment around the world, more and more governments realize that environmental pollution has become a major concern due to its negative impacts on economic development and public health. Many developed countries have employed environmental policies to alleviate pollution problems, such as the EU Clean Air Directives, the US Clean Air Act Amendments and Canada Wide Standards for Particulate Matter and Ozone. These stricter pollution regulations in turn affect the plant location and trade flows, which is called “pollution haven effect” (PHE) by Copeland and Taylor [1].

**Figure 1:**
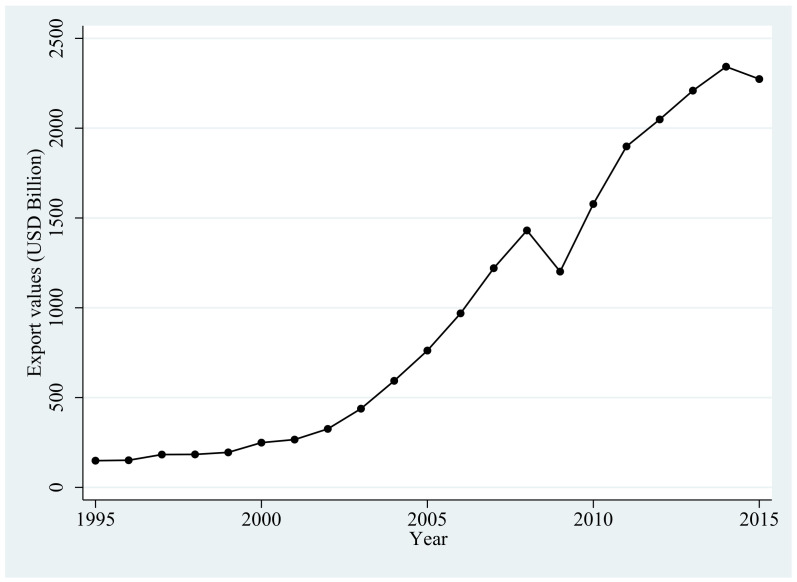
Export values in China between 1995 and 2015.

Moreover, Copeland and Taylor [1] propose a “pollution haven hypothesis” which suggests that the decrease in trade barriers will cause pollution-intensive industries from countries with stricter regulations to move to countries with lax environmental regulations. Joining the World Trade Organization (WTO) is one of the important measures for a country to reduce trade barriers. In recent years, many studies conduct a series of analysis based on China’s accession to the WTO in December 2001 [2,3,4,5,6,7]. China’s exports witnessed a dramatic increase after China’s WTO accession due to the decline in trade barriers. Figure 1 show the surge in Chinese exports (Current price) between 1995 and 2015.

The increase in Chinese exports has affected pollution emissions and health [8]. Although the increase in exports promotes Chinese economic development, the environmental problems become increasingly severe due to China’s status as the world’s factory. Before China’s accession to the WTO, the Chinese government has employed some environmental policies to alleviate pollution problems since the ninth Five-Year Plan from 1996 to 2000, but the policies were not implemented well. It is from the eleventh Five-Year Plan in 2006 that the Chinese government started to adopt more stringent measures than before. Recent studies explore the impacts of environmental regulation in the Five-Year Plan in China [9,10,11,12,13]. Among these studies, Shi and Xu [9] exploit province-level SO_2_-related environmental policy set by China’s eleventh Five-Year Plan to investigate the effect of environmental regulation on exports.

Compared with province-level SO_2_-related environmental policy used by Shi and Xu [9] which set SO_2_ reduction targets for each province, we use city-level air-related environmental policy in China’s twelfth Five-Year Plan to examine the impact of environmental regulation on exports. This air-related environmental policy is China’s first comprehensive air pollution prevention and control plan, indicating that the direction of air pollution prevention and control in China has gradually shifted from controlling total pollutant emissions to improving environmental quality. Importantly, this environmental policy specified clear criteria on how to designate some cities to set stricter environmental regulations, which largely solves the potential endogenous problem in the empirical analysis. Moreover, we exploit the Chinese Custom Database which covers all trade transactions at the Harmonization System (HS) 8-digit product level and corresponding firm information, while China’s Industrial Enterprises Database used by Shi and Xu [9] only covers state-owned enterprises (SOE) and non-SOEs with sales above 5 million RMB (roughly $ 827,000). In addition, Shi and Xu [9] explored the potential channel by examining if the environmental policy affects whether an incumbent exporter exports and a non-exporter enters the export marker, while our study examines the potential mechanism based on changes in the number of firms and export values in the city caused by firm entry and exit. Therefore, in terms of environmental policy, causal identification, data usage, and mechanism discussion, this study is different from Shi and Xu [9], thus further enriching existing studies regarding the environment and exports.

The main finding of this study is that the impact of stringent environmental regulation on exports is negative and statistically significant, this result is robust against a series of robustness checks, including the anticipation effect, the parallel trend assumption, two-period estimation, dropping special observations, alternative measure of the dependent variable, and controlling for simultaneous policies. Moreover, the decline in exports in cities with stricter environmental policies is mainly because the decline in export values caused by the exit of exporting firms exceeds the increase in export values caused by new entrants, rather than the change in the number of exporting firms in the city. In addition, we explore the heterogeneous impacts of environmental regulation on exports based on the specific differences of environmental policy we are concerned about across cities and the Broad Economic Categories (BEC) classification of products.

The remainder of the paper is structured as follows. Section 2 is the literature review. Section 3 introduces the policy background. Section 4 describes the empirical strategy and data sources. Section 5 discusses our main results and reports a series of robustness checks, as well as explores the potential mechanism and heterogeneous effects. Section 6 concludes the paper.

## 2. Literature Review

This study primarily contributes to two strands of literature related to the PHE. The first is closely related to the literature investigating the impact of environmental regulation on exports. Cherniwchan and Najjar [14] employ Canadian plant-level data to investigate the relationship between environmental regulation and export volumes and the likelihood that plants exit exporting. In addition to Shi and Xu [9] mentioned above, Hering and Poncet [15], and Zhang et al. [16] also examine the effect of environmental regulation on exports in the context of China. The main differences between these two studies and our study are that Hering and Poncet [15] do not explore the potential mechanism though which environmental regulation affects exports, and Zhang et al. [16] exploit water pollutant related environmental policy to examine the effect of environmental regulation in the textile industry. Furthermore, based on these studies regarding environmental policy and exports, because environmental regulation can bring extra costs to firms, we propose a hypothesis that the effect of the comprehensive air pollution prevention and control plan on exports may be negative.

The second strand is linked to the literature on the effect of environmental regulation on foreign direct investment. Dean et al. [17], Hanna [18], Millimet and Roy [19], and Cai et al. [20] investigate the impact of stricter environmental regulation on foreign direct investment. Specifically, based on US data, Hanna [18] finds firms with heavier regulation fail to significantly increase foreign investment in developing countries using firm-level data, Millimet and Roy [19] find environmental regulation impedes the inflow of foreign direct investment using state-level data; based on Chinese firm-level data, Dean et al. [17] show that weak environmental standards mainly attract equity joint ventures in highly-polluting industries funded through Hong Kong, Macao, and Taiwan, not significantly attract equity joint ventures from non-ethnically Chinese sources. Cai et al. [20] find that stricter environmental regulation leads to less foreign direct investment.

## 3. Policy Background

For Chinese economic and social development, the Five-Year Plan is a very important policy which contains a series of detailed plans or targets in different aspects, including economic growth, education, employment, social security, etc. The first Five-Year Plan was established in 1953, and economic growth has always been a primary task of the Five-Year Plan. It was not until the ninth Five-Year Plan from 1996 to 2000 that environmental issues started to be considered. However, the emissions reduction goal set by the ninth Five-Year Plan was not accomplished. And in the subsequent tenth Five-Year Plan from 2001 to 2005, environmentally related targets were not still accomplished. Specifically, compared with 2000, SO_2_ emissions in 2005 increased by 27.8%, the decrease in chemical oxygen demand (COD) emissions failed to achieve the pollution control target set by the tenth Five-Year Plan.

As the pollution problem aggravated, the Chinese government started to formulate stricter environmental regulation in the eleventh Five-Year Plan from 2006 to 2010, and firstly decomposed the overall national goal to each province. Moreover, the performance of government officers in terms of environmental governance was evaluated and directly related to their promotion. The eleventh Five-Year Plan is a turning point in the effectiveness of environmental governance [9,10].

Compared with 2005, SO_2_ emissions in 2010 decreased by14.29% which exceeded the emission reduction target set by the eleventh Five-Year Plan. However, the emissions of major air pollutants in China were still huge in 2010. Specifically, the total emissions of SO_2_ and nitrogen oxides were 22.68 million tons and 22.74 million tons respectively, ranking first in the world, and the emissions of soot (dust) were 14.46 million tons, which greatly exceeded the environmental carrying capacity. In addition to continuing to set SO_2_ emission reduction targets, the twelfth Five-Year Plan further set emission reduction targets for ammonia nitrogen and nitrogen oxides. Moreover, as Chinese government realized that regional complex atmospheric environmental problems have brought huge challenges to environmental governance, for the long-term sustainable economic development and people’s health, the Air Pollution Control in Key Zones (APCKZ) of the twelfth Five-Year Plan was approved by the State Council in September 2012, and then announced in December 2012. The APCKZ policy is China’s first comprehensive air pollution prevention and control plan. In this policy, the 13 zones where the level of economic development and pollution emissions were highly concentrated, as well as where the atmospheric environmental problems were more severe, were designated as the key zones. In detail, the 13 key zones accounted for about 14% of the nation’s territory, 48% of the population, 71% of GDP, 52% of total coal consumption, 48% of total SO_2_ emissions, 51% of total nitrogen oxides, 42% of soot (dust), and about 50% of Volatile Organic Compounds in 2010. The intensity of pollutant emissions per unit area was also 2.9 to 3.6 times the national average.

Based on geographical features, economic development levels, air pollution degrees, urban spatial distribution, and the flow pattern of atmospheric pollutants, the key zones were further divided into key control zones (KCZ) and ordinary control zones (OCZ) with differential environmental criteria. There were 47 cities designated as KCZ cities which were set with stricter environmental access conditions, special emission limits for pollutants in key industries, and stronger pollution control measures, and the remaining cities in key zones were designated as OCZ cities.

## 4. Empirical Strategy and Data

### 4.1. Empirical Strategy

Because the government implemented stricter environmental regulations for KCZ cities, we can investigate the impact of APCKZ policy on export values by using a difference-in-differences (DID) method. Specifically, we use the time variation (i.e., before and after the start of the APCKZ policy), and different environmental regulation criteria (i.e., KCZ cities versus other cities). The DID estimation specification is as follows:

(1) $$lnExport_{ct}=\beta KCZ_{c}\times Post2013_{t}+X_{c}^{\prime }\gamma +\delta_{c}+\delta_{t}+\varepsilon_{ct}$$

where *c* and *t* denote the city and year, respectively; the dependent variable, $lnExport_{ct}$, is the natural logarithm form of the export values at year *t* for city *c*. The first term of the second member is the DID term of interest, $KCZ_{c}$ and $Post_{t}$ are dummy variables, $KCZ_{c}$ equals to 1 for KCZ cities, and 0 otherwise, $Post2013_{t}$ equals to 1 for the years from 2013 afterward and 0 otherwise. The second term of the second member, $X_{c}^{\prime }$, is a vector including interactions of year dummies and time-invariant city characteristics in the initial year (2010) of research sampling. Because the APCKZ policy designated a city as a KCZ city based on geographical features, the level of economic development and air pollution, urban spatial distribution, and the flow pattern of atmospheric pollutants within a city, combining the available data, we use gross domestic product (GDP), GDP per capita, the emissions of SO_2_, and industrial soot (dust) to construct the interaction terms with year dummies. The rest of selection criteria for KCZ cities are included in the fixed effects. $\delta_{c}$ represents the city fixed effect controlling for all time-invariant city characteristics, such as geographical features, urban spatial distribution, and the flow pattern of atmospheric pollutants within a city. $\delta_{t}$ represents the year fixed effect capturing common shocks affecting all the cities, and $\varepsilon_{ct}$ represents the error term. Standard errors are clustered at the province level to deal with potential heteroskedasticity and serial correlation.

**Figure 2:**
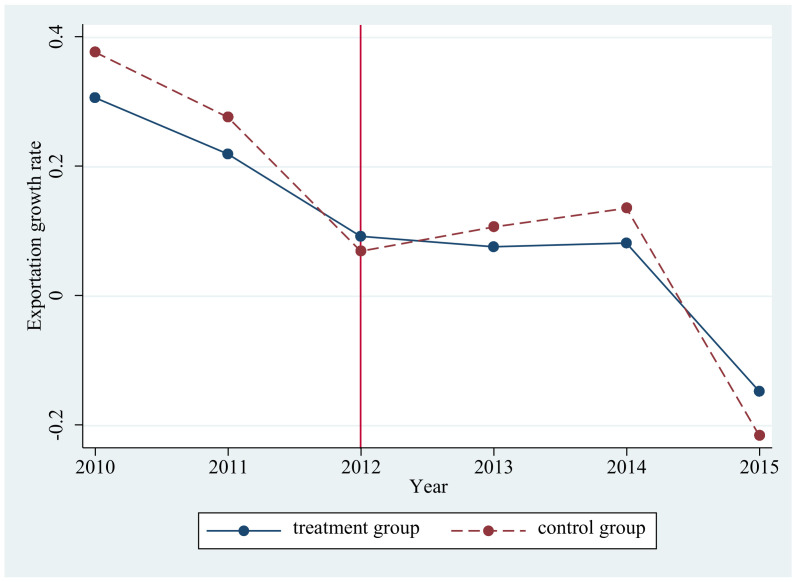
Exportation growth rate between the treatment and control groups.

Moreover, to validate the assumption of parallel path, we compare KCZ cities (the treatment group) and other cities (the control group) in terms of the mean of exportation growth rate in Figure 2. Clearly the treatment group and the control group are comparable before the implementation of the APCKZ policy, while they diverge in the post-APCKZ policy period.

### 4.2. Data

The main data in this study is the Chinese Custom Database collected by the Chinese General Administration of Customs, from 2010 to 2015. This dataset covers the universe of Chinese import and export transactions at the HS 8-digit product level. For each trade transaction, the Chinese Custom Database includes the values, quantity, quantity units of import or export, source or destination countries, and related firm information such as firms’ name, location, and many others. We primarily use it to measure the import values and export values at the city level. Another data is the China City Statistical Yearbook during the same period, which provides a lot of city information in China such as economy, population, and employment. In addition, we take advantage of many official documents to determine which cities are KCZ cities or OCZ cities, and obtain various pollutant emissions reduction targets, including SO_2_, COD, ammonia nitrogen, and nitrogen oxides. The summary statistics of the main variables are reported in Table 1.

## 5. Empirical Results and Discussion

### 5.1. Baseline Results

Our baseline estimation examined the impact of the APCKZ policy on exports. Based on Equation (1), the results are reported in Table 2. For all regressions, we included year fixed effects and city fixed effects. From Column (1) to Column (4), we stepwise included the determinants of designating KCZ cities, including the interactions between year dummies and GDP, GDP per capita, the emissions of SO_2_, and industrial soot (dust). The estimation coefficients of $KCZ_{c}\times Post2013_{t}$ remained negative and statistically significant, suggesting that stricter air pollution regulation leads to a relative reduction in exports of KCZ cities. With the increase in air pollution regulation for KCZ cities, firms located in KCZ cities face extra production costs, thus leading to the relative decrease in exports. Moreover, this effect is temporary because the policy sets pollution reduction targets for cities for 2015 and firms may take some measures to alleviate the negative impact of stricter environmental regulation such as purchasing pollution control devices or updating production technology.

### 5.2. Robustness Checks

#### 5.2.1. Expectation Effect

To confirm the exogenous assumption of the policy implementation, we checked whether cities had expected the APCKZ policy. If so, cities then adjusted related policies prior to the announcement of the official document, suggesting that our basic results might be biased. We used One Year Before 2013, equaling to 1 if the year is in 2012 and 0 otherwise, to represent a pre-policy variable. In Column (1) of Table 3, we then added an interaction term of KCZ and One Year Before 2013, namely $KCZ_{c}\times Year2012_{t}$.The coefficient of $KCZ_{c}\times Post2013_{t}$ remained negative and statistically significant, while the coefficient of $KCZ_{c}\times Year2012_{t}$ was not statistically significant, suggesting little expectation effect.

#### 5.2.2. The Parallel Trend Assumption

The most important precondition for the validity of the DID specification is to satisfy the parallel trend assumption, ensuring a similar trend between the treatment and control groups for the pre-shock period. In our baseline results, we used a year dummy $Post2013_{t}$ to construct the interaction term of interest, which meant that the estimation of baseline results yielded the average treatment effects by comparing mean differences in export values between KCZ cities and other cities, and between the pre- and post- APCKZ policy periods. To verify the parallel trend assumption, we therefore constructed a dynamic DID model as follows:

(2) $$lnExport_{ct}=\beta_{t}\sum_{t=2011}^{2015}KCZ_{c}\times Year_{t}+X_{c}^{\prime }\gamma +\delta_{c}+\delta_{t}+\varepsilon_{ct}$$

Compared with Equation (1), we replaced $KCZ_{c}\times Post2013_{t}$ with interactions of the $KCZ_{c}$ and the full set of year dummies. Based on Equation (2), the result in Column (2) shows the estimated coefficients were insignificant before 2013, and the interactions turned negative and significant from 2013, which suggests that the parallel trend assumption is satisfied and further validates our main empirical strategy.

#### 5.2.3. Two-Period Estimation

To alleviate the serial correlation of error terms, we previously clustered the error term at the province level. As a robustness check, we next used an alternative approach to collapse the data into two periods (pre- and post- APCKZ policy), as suggested by Bertrand et al. [21], to help reduce serial correlation. The results are presented in Column (3), with qualitatively similar results.

#### 5.2.4. Drop Observations of Chongqing

Different from other KCZ cities, only the urban districts of Chongqing belong to the KCZ. In order to confirm whether the basic results were reliable, we dropped observations of Chongqing in Column (4). The estimation result remained robust.

#### 5.2.5. Alternative Measure of the Dependent Variable

To alleviate the impact of the shared time-trend which may lead to the high R-Square, we used the change in export values between t − 1 and t as the dependent variable to regress in Column (5). The results show that the R-Square became lower, and importantly, the main result remained negative and statistically significant.

#### 5.2.6. Other Simultaneous Policies

Our estimation may be contaminated by simultaneous policies which have different impacts on the treatment and the control groups of this study. An important consideration that may affect our results is other environmental policies in the twelfth Five-Year Plan which set the provincial pollution reduction targets in 2012 for the emissions of CO_2_, nitrogen oxides, SO_2_, ammonia nitrogen, and COD. We therefore added interaction items of the pollution reduction targets and $Post2012_{t}$ to control for these reforms. The regression results are reported in Table 4, which shows that the coefficients of $KCZ_{c}\times Post2013_{t}$ remained negative and statistically significant. Hence, we can rule out the possibility that our results are caused by simultaneous environmental policies.

### 5.3. Mechanism Discussion

In this section, we explore the possible mechanism through which the APCKZ policy affects export values. We firstly investigated the impact of APCKZ policy on the number of firms in the city. The result in Column (1) of Table 5 shows that the APCKZ policy did not have a significant effect on the number of firms in KCZ cities, suggesting that the negative effect of APCKZ policy on exports is not because a large number of firms exit the export market in KCZ cities. Next, we investigated the potential mechanism from the perspective of export values based on firms’ export status. We defined firms as new entrants, incumbents, and exiters, and then obtained the corresponding export values in each city. Specifically, we defined new entrants as firms that never exported before the APCKZ policy and started to export after the APCKZ policy, exiters as firms that exported before the APCKZ policy and never exported after the APCKZ policy, and incumbents as firms that exported before and after the APCKZ policy. The results in Columns (2)–(4) of Table 5 show that the impact of APCKZ policy on exports of new entrants was significantly positive, while the impact on exports of incumbents and exiters were both significantly negative. In terms of coefficient size, we found that although the absolute value of estimation coefficient for new entrants was larger than that for incumbents, it was less than that for exiters, which means that the APCKZ policy led to the decrease in export values for KCZ cities as a whole.

### 5.4. Heterogeneity Discussion

We have investigated the impact of APCKZ policy on exports by comparing KCZ cities with the rest of cities. However, the rest of cities include OCZ cities and other cities which are not included in key zones of APCKZ policy. In order to investigate the impact of environmental regulation on exports between KCZ cities and these two types of cities, we restricted the sample to KCZ cities and OCZ cities in Column (1) of Table 6, and the sample to KCZ cities and other cities which are not controlled by APCKZ policy in Column (2) of Table 6. The results show that the coefficient of $KCZ_{c}\times Post2013_{t}$ in Column (1) was not statistically significant, while that of $KCZ_{c}\times Post2013_{t}$ in Column (2) was statistically significant and negative. These findings suggest that compared with KCZ cities, the exports of OCZ cities also decreased due to APCKZ policy, while the exports of other cities that are not controlled by APCKZ policy were not significantly negatively affected.

Based on the BEC classification and conversion table between BEC and HS code, we measured the exports and imports of intermediate goods and consumption goods. The dependent variables in Columns (3)–(4) of Table 6 are the natural logarithm form of exports of intermediate goods and consumption goods, and the dependent variables in Columns (5)–(6) are the natural logarithm form of the imports of intermediate goods and consumption goods. We found that the APCKZ policy had a statistically significant and negative impact on exports and imports of intermediate goods, and had no impact on exports and imports of consumption goods. The possible reason is that a lot of high-polluting intermediate production processes are carried out in China due to its characterization as the world’s factory, which in turn leads to the significant decline in exports and imports of intermediate goods under stricter environmental regulations.

## 6. Conclusions

Compared with environmental policies regarding the pollution control target in previous studies in the context of China, the APCKZ policy is a comprehensive air pollution prevention and control plan. There is little evidence on the impact of APCKZ policy. Taking advantage of the APCKZ policy as a quasi-natural experiment, this study exploited the DID estimation to investigate the causal relationship between environmental regulation and export values based on the Chinese Custom Database and China City Statistical Yearbook. Moreover, we explored the possible mechanisms and heterogeneous effects of environmental regulation.

We found that stricter environmental regulation has a negative and statistically significant impact on export values. This main conclusion remains robust against a series of robustness checks, such as expectation effect, the parallel trend assumption, two-period estimation, etc. Furthermore, we explored the potential mechanism based on firms’ entry and exit, and found that the potential mechanism through which the APCKZ policy affects cities’ export values may be the change in the export values caused by firm entry and exit, especially by exiters, rather than the change in the numbers of firms in the city caused by firm entry and exit. Finally, our heterogeneous analysis showed that the exports of OCZ cities were also negatively affected by the APCKZ policy compared to KCZ cities, while the exports of cities not included in the key zones of APCKZ policy were not significantly affected. And the negative effect of APCKZ policy on exports was greater and more significant for intermediate goods.

Moreover, our results can provide important policy implications. Nowadays, not only for developed countries, but also developing countries like China, it is very important that they reduce pollution and protect the environment. More and more countries have started to implement environmental policies. Although environmental policy is beneficial in many ways, in terms of the economy and public health, our study finds a negative effect of stringent environmental regulation on exports. And this negative regulatory impact comes from firm entry and exit, especially by exiters. Furthermore, the exports of cities that are not directly affected by environmental regulation are not significantly affected; the exports of intermediate goods are more negatively affected. Policymakers therefore need to take some measures to alleviate the negative impact on exports in future policymaking, for example, giving appropriate subsidies for firms to purchase pollution control devices and update pollution abatement technologies, paying attention to the environmental quality of cities not directly affected by environmental policy to avoid pollution transfer, and providing some preferential measures for trade in intermediate goods. As a result, our findings are helpful for the construction of an accurate policy package to alleviate the negative impacts of stricter controls on export values. However, it is a pity that the model in this study is static because the policy does not provide immediate effects. We hope that future research will break through this limitation by using new methods.

## Figures and Tables

**Table 1:** Summary statistics.

Variable	Unit	Observations	Mean	SD
Export	US dollar	1656	20.08	2.247
GDP	US dollar	1656	23.38	0.887
GDP Per Capita	US dollar	1656	8.322	0.596
SO_2_ Emissions	Ton	1656	10.60	1.080
Soot (dust) Emissions	Ton	1656	9.436	1.057

Note: The summary statistics in Table 1 is based on the natural logarithm form of the variables; GDP is the abbreviation for gross domestic product.

**Table 2:** Baseline Results.

Variable	(1)	(2)	(3)	(4)
$KCZ_{c}\times Post2013_{t}$	−0.187 **	−0.196 **	−0.188 **	−0.183 **
	(0.0763)	(0.0749)	(0.0736)	(0.0712)
$Post2013_{t}$	−2.617	−3.175	−2.760	−2.789
	(2.4354)	(2.3496)	(2.2682)	(2.2620)
Year Fixed Effect	Yes	Yes	Yes	Yes
City Fixed Effect	Yes	Yes	Yes	Yes
GDP × year dummies	Yes	Yes	Yes	Yes
GDP Per Capita × year dummies	No	Yes	Yes	Yes
SO_2_ Emissions × year dummies	No	No	Yes	Yes
Soot (dust) Emissions × year dummies	No	No	No	Yes
R-squared	0.961	0.961	0.961	0.961
Observations	1680	1668	1656	1656

Note: Standard errors in parentheses are clustered at the province level. ** indicate significance at the 5% levels, respectively; GDP is the abbreviation for gross domestic product.

**Table 3:** Robustness checks.

Variable	(1)	(2)	(3)	(4)	(5)
$KCZ_{c}\times Post2013_{t}$	−0.205 **		−0.189 ***	−0.198 ***	−0.082 **
	(0.0788)		(0.0649)	(0.0695)	(0.039)
$KCZ_{c}\times Year2011_{t}$		0.00898			
		(0.0652)			
$KCZ_{c}\times Year2012_{t}$	−0.0658	−0.0612			
	(0.0614)	(0.0663)			
$KCZ_{c}\times Year2013_{t}\;$		−0.141 *			
		(0.0717)			
$KCZ_{c}\times Year2014_{t}$		−0.188 *			
		(0.0935)			
$KCZ_{c}\times Year2015_{t}\;$		−0.274 **			
		(0.1145)			
Controls	Yes	Yes	Yes	Yes	Yes
Year Fixed Effect	Yes	Yes	Yes	Yes	Yes
City Fixed Effect	Yes	Yes	Yes	Yes	Yes
R-squared	0.962	0.962	0.976	0.961	0.323
Observations	1656	1656	554	1650	1379

Note: Standard errors in parentheses are clustered at the province level. ***, **, and * indicate significance at the 1%, 5%, and 10% levels, respectively. Controls include $Post2013_{t}$, GDP × year dummies, GDP Per Capita × year dummies, SO_2_ Emissions × year dummies, and Soot (dust) Emissions × year dummies.

**Table 4:** Other simultaneous policies.

Variable	(1)	(2)	(3)	(4)	(5)	(6)
$KCZ_{c}\times Post2013_{t}$	−0.182 **	−0.181 **	−0.184 **	−0.182 **	−0.178 **	−0.172 **
	(0.0689)	(0.0695)	(0.0701)	(0.0709)	(0.0699)	(0.0721)
CO_2_ nitrogen oxides	Yes					Yes
		Yes				Yes
SO_2_ ammonia nitrogen			Yes			Yes
				Yes		Yes
COD					Yes	Yes
Controls	Yes	Yes	Yes	Yes	Yes	Yes
Year Fixed Effect	Yes	Yes	Yes	Yes	Yes	Yes
City Fixed Effect	Yes	Yes	Yes	Yes	Yes	Yes
R-squared	0.961	0.962	0.961	0.962	0.962	0.962
Observations	1656	1656	1656	1656	1656	1656

Note: Standard errors in parentheses are clustered at the province level. ** indicates significance at the 5% level, respectively. Controls include $Post2013_{t}$, GDP × year dummies, GDP Per Capita × year dummies, SO_2_ Emissions × year dummies, and Soot (dust) Emissions × year dummies. CO_2_, nitrogen oxides, SO_2_, ammonia nitrogen, and COD represent interaction items of the corresponding pollution reduction targets and $Post2012_{t}$.

**Table 5:** Mechanism discussion.

Variable	Number	New Entrants	Incumbents	Exiters
	(1)	(2)	(3)	(4)
$KCZ_{c}\times Post2013_{t}$	−0.0208	0.700 *	−0.260 **	−1.256 ***
	(0.0288)	(0.3595)	(0.1019)	(0.4322)
Controls	Yes	Yes	Yes	Yes
Year Fixed Effect	Yes	Yes	Yes	Yes
City Fixed Effect	Yes	Yes	Yes	Yes
R-squared	0.992	0.958	0.978	0.959
Observations	1656	1656	1656	1656

Note: Standard errors in parentheses are clustered at the province level. ***, **, and * indicate significance at the 1%, 5%, and 10% levels, respectively. Controls include $Post2013_{t}$, GDP × year dummies, GDP Per Capita × year dummies, SO_2_ Emissions × year dummies, and Soot (dust) Emissions × year dummies.

**Table 6:** Heterogeneity discussion.

Variable	(1)	(2)	(3)	(4)	(5)	(6)
$KCZ_{c}\times Post2013_{t}$	−0.0139	−0.236 **	−0.166 *	−0.0146	−0.245 **	−0.197
	(0.0674)	(0.0925)	(0.0851)	(0.1041)	(0.1165)	(0.2415)
Controls	Yes	Yes	Yes	Yes	Yes	Yes
Year Fixed Effect	Yes	Yes	Yes	Yes	Yes	Yes
City Fixed Effect	Yes	Yes	Yes	Yes	Yes	Yes
R-squared	0.986	0.959	0.928	0.869	0.912	0.844
Observations	688	1244	1656	1656	1626	1626

Note: Standard errors in parentheses are clustered at the province level. ** and * indicate significance at the 5% and 10% levels, respectively. Controls include $Post2013_{t}$, GDP × year dummies, GDP Per Capita × year dummies, SO_2_ Emissions × year dummies, and Soot (dust) Emissions × year dummies.

## Data Availability

Data available on request due to restrictions e.g., privacy or ethical.
